# Case Report: Rectal perforation during CT colonography

**DOI:** 10.12688/f1000research.8242.1

**Published:** 2016-03-08

**Authors:** Marianna Zukiwskyj, Yasser Arafat

**Affiliations:** 1 Department of Surgery, Rockhampton Hospital, Rockhampton, Australia; 2 Department of Surgery, Caboolture Hospital, Caboolture, Australia

**Keywords:** Rectal perforation, CT Colonography, CTC

## Abstract

Introduction

Computer tomography colonoscopy (CTC) is an increasingly prevalent procedure for the investigation of colorectal symptoms, or as a component of colorectal cancer screening.  It is considered a low risk procedure, however colonic perforation is a recognized significant complication.

Case Report

We report the case of an 81-year-old female patient who underwent CTC after failed optical colonoscopy as part of routine colorectal cancer screening.  Perforation of the rectum with surrounding pararectal air was confirmed on CTC.  The patient had minimal symptoms and was treated successful non-operatively with bowel rest and antibiotics.

Conclusion

Perforation sustained during CTC is an uncommon complication.  The incidence of perforation during CTC is still lower than that during optical colonoscopy.  In the absence of significant abdominal signs and symptoms, this rare complication may be successfully managed non-operatively.

## Introduction

The incidence of colorectal cancer worldwide has been reported as 9% ^1^. It is the third most commonly diagnosed cancer worldwide ^1^, and, excluding cutaneous cancers, is the mostly commonly diagnosed cancer in Australia ^2^. National screening programmes are an important tool for the early detection of, and effective reduction in mortality from colorectal cancer ^2, 3^. Since its inception in 1994, computer tomography colonoscopy (CTC) has been increasingly utilized for both colorectal cancer screening and investigation of colorectal symptoms ^4^. Indications for CTC include colorectal cancer screening, incomplete or failed optical colonoscopy, symptomatic or asymptomatic individuals who may have significant medical comorbidities considered high risk for optical colonoscopy. Advantages of CTC include minimal invasiveness, better patient tolerance, unlikely need for sedation, low incident of adverse events, and the occasional discovery of extra colonic pathology ^5, 6^. Colonic perforation is a significant albeit rare complication of CTC ^7^.

## Case report

**Figure 1.**
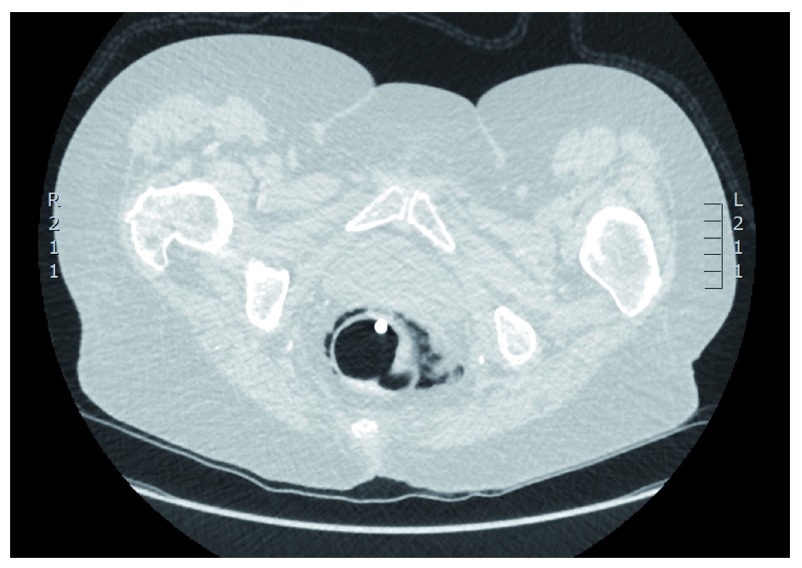
CT colonography depicting contained rectal perforation. The Foley catheter tip is seen in rectum as well as localized peri-rectal air.

**Figure 2.**
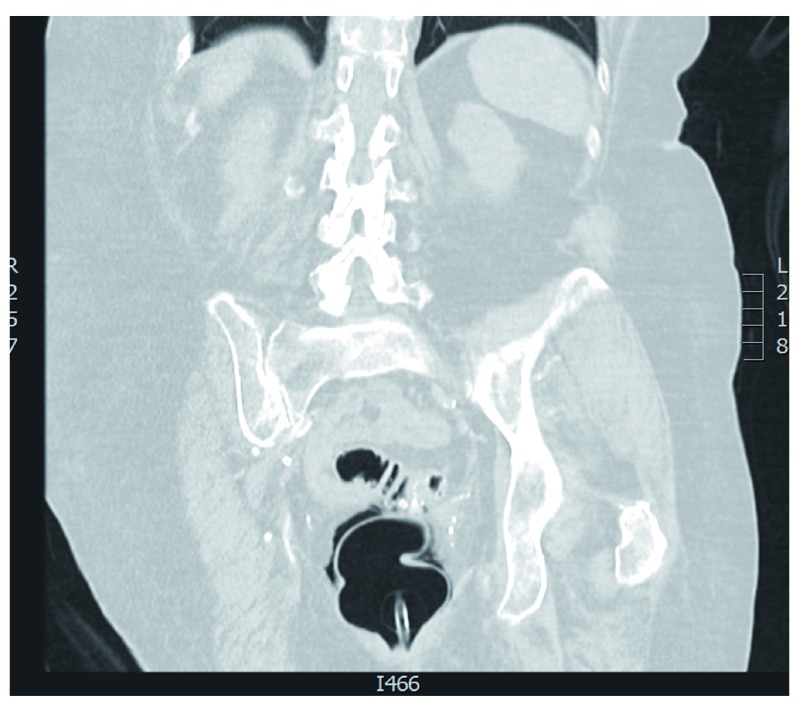
CT colonography coronal view depicting rectal perforation. The Foley catheter is seen within the rectum as well as peri-rectal air.

**Figure 3.**
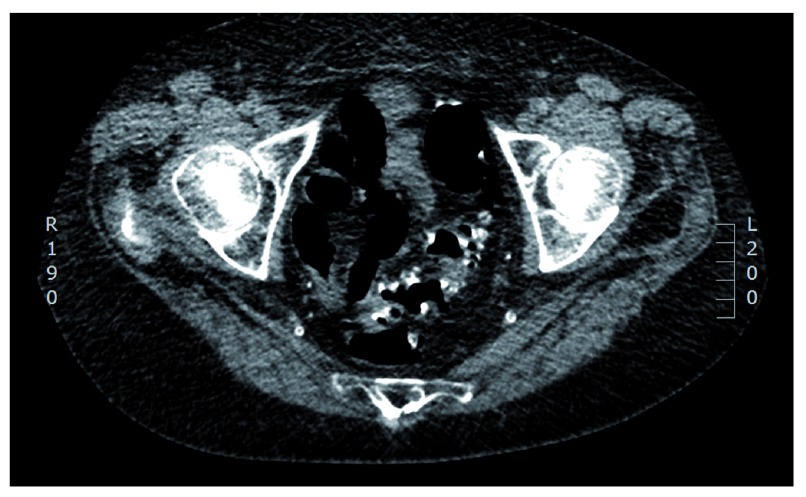
CT colonography depicting extensive diverticular disease and narrowing of the distal sigmoid.

An 81-year-old Caucasian female underwent a CTC for colorectal cancer screening. She tested positive to a faecal occult blood stool test, and had experienced longstanding, infrequent, minimal and painless bright red rectal bleeding for a period of over twenty years. She gave no history of loss of weight, change in bowel habits, or family history of colorectal cancer. Past medical history was significant for atrial fibrillation requiring anticoagulation, diverticular disease and a hysterectomy 20 years prior with subsequent radiotherapy to the pelvis as histology had confirmed uterine cancer. In the last twenty years, four screening colonoscopies had been carried out, the last five years prior, and whilst clear of polyps, was significant for an asymptomatic narrowed segment of distal sigmoid which had to be traversed with a paediatric colonoscope. The patient underwent a CTC as, she had, earlier in the year, undergone a failed optical colonoscopy. CTC was carried out with manual air insufflation via a rectal soft tip Foley catheter until the patient felt slight discomfort, at which point a scout AP film was taken to ensure adequate bowel distension. It proceeded without complications. The official report noted a localized contained perforation demonstrated around the rectum ( Figure 1, Figure 2). There was extensive diverticular disease demonstrated throughout the sigmoid colon, which was markedly narrowed throughout in keeping with stricture formation, which would be consistent with previous diverticulitis or radiation treatment ( Figure 3). Further assessment of that region was difficult. The remainder of the large bowel had achieved excellent distension.

The patient was admitted to the ward and commenced on prophylactic antibiotics (Tazocin 4.5g tds), and bowel rest. She complained of mild discomfort in the lower abdominal region, and had a soft abdomen with no evidence of peritonism. During the first two days of admission, she passed a small amount of blood clot per rectum. She was discharged on day five of admission, with no abdominal signs, having undergone an MRI which confirmed no peri-rectal collection and identified no obvious perforation. A further optical colonoscopy was attempted two months later, which failed to enter the sigmoid colon, citing severe diverticular disease as the reason. The patient currently remains asymptomatic.

## Discussion

CTC is widely considered as a minimally invasive technique with a low rate of adverse events. However, various complications arising from CTC have been reported in the literature. The most significant of these is colonic perforation. Since the inception of CTC, various publications have reported rates of perforation ranging from 0.009 to 0.1% ^4, 6, 7^. This is in comparison to the rate of perforation at optical colonoscopy, which has been reported as ranging from 0.032% to 0.196% ^4^, however is commonly quoted as 0.05% to 0.1%. The first meta-analysis on the rate of colonic perforated at CTC was conducted in 2014 by Bellini *et al*., in which an overall perforation rate of 0.04% was reported ^7^. The majority of the perforations occurred in the sigmoid colon at 41%, with rectal perforations accounting for 22.2% ^7^. When the perforation rate was adjusted for symptomatic or asymptomatic individuals (those for whom CTC was purely a screening procedure), the perforation rate of symptomatic patients was 0.08%, compared with asymptomatic patients at 0.02%, and the odds ratio was reported as 19.2 ^7^. Various factors have been considered as increasing the likelihood of perforation. The use of rigid rectal catheters, CTC shortly after optical colonoscopy with biopsies, bowel containing inguinal herniae, a history of diverticular disease, CTC with manual insufflation of gas, and obstructing lesions have all been described as contributing to perforations in the literature ^4, 7– 9^. However, given the rarity of perforations, the degree of contribution of each of the factors may vary amongst the current reviews.

Once a CTC perforation is confirmed, management may include operative and non-operative measures. The majority of reported CTC perforations have been managed non-operatively. Patients clinically suitable for non-operative management receive IV fluids, antibiotics and bowel rest. Bellini *et al.* reports that 68% of perforated patients were successful managed non-operatively ^7^.

In this case, our patient, whilst asymptomatic of colorectal symptoms, did have several risk factors for perforation. There was a history of diverticular disease and of a narrowing in the region of the sigmoid, which did not require intervention at the time of her previous colonoscopy five years prior. Manual rather than automatic insufflation was employed. A soft tipped catheter was employed, and whilst this does lessen the risk of traumatic injury, it does not negate it, as the integrity of the rectal mucosa as well as technique of insertion are factors. As the diverticular disease and stricture extended to the distal sigmoid, it could be inferred that, in this case, the site more likely at risk of perforation would be rectal, rather than sigmoid. The most recent optical colonoscopy had been six months prior and so was unlikely to be a factor.

## Conclusion

Colonoscopy is considered the gold standard for intraluminal evaluation of the colon in a variety of settings. CT colonography is an accepted alternative to optical colonoscopy in the event of failed endoscopic evaluation, as a screening procedure and in high risk candidates. The incidence of perforation at CTC is low. Bellini *et al*. reported fewer than 40 cases in their meta-analysis. As the majority of CTC perforations are managed non-operatively, the rate of CTC related surgical intervention was 0.008% ^7^. Whilst the rate of perforation is accepted as lower than that of optical colonoscopy, the more significant advantage seems to be the much higher incidence of successful non-operative management of these patients ^7, 10^.

## Consent

Written informed consent for publication of their clinical details and clinical images was obtained from the patient.
