# Supplementary material for: Case Report: Rectal perforation during CT colonography
Source: F1000Res. 2016 Mar 8;5:299. [Version 1] doi: 10.12688/f1000research.8242.1 (PMC4805160; doi:10.12688/f1000research.8242.1)
Supplement: Peer review history

# Peer review: Referee response for version 1

**DOI:** 10.5256/f1000research.8864.r12800

**Published:** 2016-03-21

**Authors:** Baljit Singh

**Affiliations:** 1 Department of Colorectal Surgery, University Hospitals of Leicester NHS Trust, Leicester, UK

A well written and interesting report. This highlights whilst this is a common procedure there is a small associated risk. However the authors suggest that in most cases this can be managed conservatively.

I have read this submission. I believe that I have an appropriate level of expertise to confirm that it is of an acceptable scientific standard.

# Peer review: Referee response for version 1

**DOI:** 10.5256/f1000research.8864.r12798

**Published:** 2016-03-21

**Authors:** Trevor Winter

**Affiliations:** 1 Kaiser Permanente, Bakersfield, CA, USA

Computer tomography colography (CTC) has been used since 1994 for colorectal screening and investigation of colorectal symptoms mainly in patients who have had incomplete optical colonoscopy, or who have significant medial comorbidities considered at high risk for optical colonoscopy. CTC is generally considered a minimally invasive procedure.  

This paper is a useful addition to the literature, highlighting the low complication rate of CTC, with a perforation rate of 0.009% to 0.1%, compared to the complication rate of optical colonoscopy of  0.032% to 0.19%.  The paper also highlights the factors associated with perforation as use of rigid rectal catheters, manual insufflation of gas CTC shortly after optical colonoscopy with biopsies, inguinal herniae, diverticular disease, and obstructing lesion. The authors comment that the majority of reported CTC perforations have been managed conservatively with IV fluids, antibiotics and bowel rest.

Although colonoscopy is still considered the gold standard, CTC is a valuable alternative in patients with incomplete optical colonoscopy or are considered to be of high risk for optical colonoscopy due to medical comorbidities.

I have read this submission. I believe that I have an appropriate level of expertise to confirm that it is of an acceptable scientific standard.
